# Cyber victimization during the COVID‐19 pandemic: A syndemic looming large

**DOI:** 10.1002/hsr2.528

**Published:** 2022-02-17

**Authors:** Sheikh Shoib, Sharad Philip, Seema Bista, Fahimeh Saeed, Sana Javed, Dorottya Ori, Adil Bashir, Miyuru Chandradasa

**Affiliations:** ^1^ Department of Psychiatry Jawahar Lal Nehru Memorial Hospital Srinagar India; ^2^ Department of Psychiatry NIMHANS Bangalore India; ^3^ Division of Clinical and Translational Research Larkin Comminity Hospital System South Miami Florida United states; ^4^ Department of Psychiatry Psychosis Research Center, University of Social Welfare and Rehabilitation Sciences Tehran Iran; ^5^ Psychiatry Unit Nishtar Medical University Multan Pakistan; ^6^ Department of Mental Health Heim Pal National Pediatric Institute, and Institute of Behavioural Sciences, Semmelweis University Budapest Hungary; ^7^ Department of Social Work Kashmir University Kashmir India; ^8^ Department of Psychiatry University of Kelaniya Kelaniya Sri Lanka

In consequence of lockdown and social isolation during COVID‐19 pandemic, everyday tasks, communications, and interactions were disrupted, and the online mode remained the only way for daily activities. Many turned to the internet, social networks, online dating for companionship, and online gaming and streaming for entertainment. Therefore, online platforms such as online gaming or dating even medical services reported an increase in rate of users. Subscribers on one platform connected 42% more than prepandemic levels. All nonemergency medical services have been reoriented and made available via online platforms. Many signed up for medical service platforms, video conferencing applications, and linked health services such as online pharmacies.^1^

Demonstrably, as peoples' online presence increased, the incidence of cyber victimization shot up alarmingly. Cyber victimization is understood as the experience of aggressive behaviors while using information and communication technology modes such as the internet, gaming consoles, and smartphones.^2^ This broadly includes netizens becoming victims of violence, harassment, trolling, stalking, bullying, and crimes in the cyber world. Studies have shown that cyberbullies targeted people with physical impairments, intellectual disabilities, and chronic diseases, causing depressive symptoms, anxiety, distress, somatic health complaints, and self‐harm.^3^ Cyberbullying increased during the pandemic. However, it had a prevalence ranging up from 6% to 35% in studied samples before^4^ it increased during the pandemic. Students who preferred to use Instagram, online gaming, more number of play, also being opinionated on platforms were at higher risk of cyberbullying experience.^5^ In addition, being diagnosed with COVID‐19 or knowing infected people was associated with cyberbullying through stress.^6^ Cyberbullying was associated with the use of three or more hours of internet daily, web cameras, text messages, and posting personal information.^4^ Not only the cyber‐victims, but also the cyberbullies had more emotional dysregulation, psychosomatic problems, and social context difficulties.

Demonstrably, as peoples' online presence increased, the incidence of cyber victimization shot up alarmingly.^7^, ^8^ Cyber victimization is understood as the experience of aggressive behaviors while using information and communication technology modes such as the internet, gaming consoles, and smartphones.^2^ This broadly includes netizens becoming victims of violence, harassment, trolling, stalking, bullying, and crimes in the cyber world. Accordingly, authorities issued alerts regarding organized rackets engaging in cyber fraud, romance scams, financial scams, “doxing,” “phishing,” and many other nefarious activities.^4^ Cyber victimization also includes being at the receiving end of hurtful online activities such as harassing messages and disparaging comments and/or humiliating pictures^9^, ^10^; worst of these being threatened, intimated, and blackmailed.^11^ Throughout, the authors refer to cyber victimization as a larger paradigm of being victimized while cyberbullying refers to the perpetration of aggressive and hurtful online activities. Studies have shown that cyberbullies targeted people with physical impairments, intellectual disabilities, and chronic diseases, causing depressive symptoms, anxiety, distress, somatic health complaints, and self‐harm.^3^ It may be that even cyberbullies experience increased emotional dysregulation, psychosomatic problems, social context difficulties, and may have themselves been victims. Estimates of the incidence of cyber victimization before the pandemic ranged from 6% to 35% in studied samples.^4^ Those at higher risk included students who used Instagram, played online games, had more number of plays, and were opinionated on these platforms.^5^ Other factors included the use of three or more hours of internet daily, web cameras, text messages, and posting personal information.^4^ Furthermore, being diagnosed with COVID‐19 or knowing infected people was associated with cyber victimization.^6^

Being a victim in the cyber world is markedly different from being one in the real world. The cyber victimization is characterized by, victimizing events more widely disseminated with an unlimited reach, the abusive items may haunt as archives, help and support for cyber victims are far less proportional to the witnesses, perpetrators are virtually away from the victim without personal physical contact leading to lack of remorse, and disparity of the perceived impact between the perpetrators and victim. Contrary to popular understanding, being a cyber‐victim is just as severe, if not more, as in the face‐to‐face world.^11^ Myriad implications exist for victims' mental health, including, but not limited to, anxiety, panic symptoms, distress, trauma symptoms, sadness, hopelessness, helplessness, reduced self‐esteem, feelings of isolation, fear of socialization, suicidal ideation, self‐harm, somatic symptoms like headaches, stomach‐aches, changes in sleep, appetite, psychoactive substance use, and interpersonal difficulties.^11^, ^12^ These adverse impacts on mental health must be contextualized in the background of limited mental health care access, remote avenues of support from peers, and social networks during this pandemic. Unmitigated mental distress often exacerbates severe symptoms: even suicide attempts.^13^

Cyber victimization experiences can even lead to broadcasting death through live streaming, named cyber suicide.^14^ There has been public outcry in Japan after a reality TV actor and ex‐professional wrestler died by suicide after being cyber victimized. Access to “how to” resources‐ descriptions, including video content have become more accessible and available via video blogging and sharing platforms.^15^ Often, online chat rooms and unmoderated platforms become public for facilitating the discussions on dysphoric thoughts and feelings, which leads to the formation of cyber suicide pacts involving virtual strangers. Unassuming youth have been known to enter online challenges and their fate culminating in suicides, for example, in the blue whale challenge and choking game. Persons with mental health conditions are at greater risk of experiencing cyber victimization and more severe mental health consequences. Vulnerabilities could include cognitive disturbances, problematic internet use, impulsive behaviors, as well as memory difficulties. Irresponsible and sensationalized media reporting of suicides, even cyber suicide can lead to the amplification of the Werther effect.^3^, ^16^

As the pandemic has raged on globally, the suicide rates have not dipped despite limited access to lethal means.^17^ There are no data available to affirm whether cyber suicides have risen in proportion. Nevertheless, we posit that increasingly online life has exacerbated the cyber suicide crisis. We note that such a syndemic is either at hand or in play considering the adverse mental health consequences of cyber victimization. We propose that the definition of a syndemic be broadened to include noninfectious health consequences and cyber suicide be recorded in a disaggregated manner. With greater recognition of the issues surrounding cyber victimization, concerted efforts toward its prevention would hopefully be undertaken.

Online entertainment websites and platforms promote infinite scrolls and seek to maximize engagements with their products. They should also discharge their moral responsibility to signpost for psychological support, provide trauma care, and moderate online content. This should reflect how communities deal with aggression in the face‐to‐face world. Psychological support should be extended to the victims and witnesses. Steps should go beyond usually fruitless and oftentimes retraumatizing calls for legal action or accountability. All web platforms need to develop and advertise anti cyber victimization policies. These could include regular quizzes on the impact and surveys on whether there have been such experiences in the recent past. All platforms promoting cyber interactions ought to take up the mantle of encouraging prosocial behaviors. All health professionals especially those in mental health will do their best to educate clients and families about steps to remain cyber secure and report cyber victimization.

We hope this write‐up would alert all mental health professionals and act as a call to action by law enforcement agencies and online platforms. Every measure tenable should be taken to avert this syndemic of cyber victimization, cyber suicide, and the COVID‐19 pandemic.

## FUNDING

None declared.

## CONFLICT OF INTEREST

The authors declare that there is no conflict of interest.

## AUTHOR CONTRIBUTIONS

Conceptualization: Sheikh Shoib.

Writing Original Draft Preparation: Sheikh Shoib.

Writing, Review, & Editing: Sheikh Shoib, Sharad Philip, Seema Bista, Fahimeh Saeed, Sana Javed, Dorottya Ori, Adil Bashir, Miyuru Chandradasa.

All authors have read and approved the final version of the manuscript.

Sheikh Shoib had full access to all of the data in this study and takes complete responsibility for the integrity of the data and the accuracy of the data analysis.
